# What Does It Mean to Be a Young African Woman on a University Campus in Times of Sexual Violence? A New Moment, a New Conversation

**DOI:** 10.3390/bs8080067

**Published:** 2018-07-26

**Authors:** Astrid Treffry-Goatley, Naydene de Lange, Relebohile Moletsane, Nkonzo Mkhize, Lungile Masinga

**Affiliations:** 1 School of Education, University of KwaZulu-Natal (UKZN), Durban 3605, South Africa; 2 Faculty of Education, Nelson Mandela University, Port Elizabeth 6031, South Africa; Naydene.deLange@nmmu.ac.za; 3 School of Education, UKZN, Durban 3605, South Africa; moletsaner@ukzn.ac.za (R.M.); mkhizen2@ukzn.ac.za (N.M.); masingal@ukzn.ac.za (L.M.)

**Keywords:** sexual violence, South Africa, participatory research, participatory visual research, transformative learning theory

## Abstract

Sexual violence in the higher education is an epidemic of global proportions. Scholars conclude that the individual and collective silence that surrounds such violence enables its perpetration and that violence will only be eradicated when we break this silence. In this paper, we used two participatory visual methods (PVM), collage and storytelling, to explore what sexual violence at university looks like and what it means to woman students. Two groups of student teachers in two South African universities were engaged in collage and storytelling workshops in late 2017 and early 2018, respectively. We thematically analyzed the issues that emerged from the data, drawing on transformative learning theory to explore how our approach might help women students to break the silence around sexual violence and stimulate critical dialogue to address it. Our analysis suggests that these visual tools enabled deep reflections on the meaning and impact of sexual violence, particularly for women. In addition, the participatory process supported introspection about their experiences of sexual violence and their responses to it as bystanders in and around campus. More importantly, they discussed how they, as young women, might break the silence and sustain new conversations about gender and gender equality in institutions and beyond.

## 1. Introduction

The recent #MeToo Twitter movement of 2017 brought international attention to the prevalence of sexual violence in private and public spaces throughout the world. Defined as ‘any sexual act, attempt to obtain a sexual act, unwanted sexual comments or advances, or acts to traffic, or otherwise directed against a person’s sexuality using coercion, by any person regardless of their relationship to the victim, in any setting, including but not limited to home and work’ [1,2], sexual violence is negatively skewed against girls and women. The #MeToo movement and the subsequent campaigns on college and university campuses across the world have brought the pervasiveness of sexual violence against women to the fore in a context where voices against such violence are often silenced. In South Africa, sexual violence is rife and women and girls experience rape and other forms of gender-based violence on a ‘large and seemingly uncontrollable scale’ [3]. Yet the authorities seem incapable of responding effectively to the situation [3]. For example, while over 40,000 rapes are reported to the police per annum [4], it is estimated that only 14% of perpetrators face conviction in South Africa [5]. In fact, evidence suggests that statistics are much higher, since fear, shame, re-traumatization, and a lack of trust in the criminal justice system discourage women and girls from reporting sexual violence [6]. Consequently, just one in nine cases of sexual violence ever reach police records [6,7,8]. The pervasiveness of rape and other forms of sexual violence against women and girls in South Africa, and the inadequacy of the state’s response, have fostered a ‘rape culture’ in which sexual violence has become normalized. Rape culture goes beyond the physical act of rape or sexual abuse. It is a mindset, created and enabled by patriarchy that serves to empower men at the expense of women, keeping women and men within ‘certain boundaries and categories’ [9]. Rape culture effectively undermines the citizenship of women and girls [3], demoting them to ‘second class citizens’ and serving to control their behaviour through a culture of silence, fear, and shame. Our objective in this study was to explore the nature of sexual violence on campus and to gain insight into what it means to be an African woman student in times of sexual violence.

## 2. Sexual Violence on Higher Education Campuses

Sexual violence in the higher education sector has been a worldwide concern for many decades [10,11,12,13]. It has far-reaching public health, economic and social consequences. For example, students with a history of being sexually violated are prone to depression, anxiety, and headaches and are less likely to perform well academically [14,15]. In addition, since most sexual violence occurs within the context of intimate partner relationships, survivors are known to suffer from the trauma of interpersonal betrayal [16]. Yet when sexual violence occurs within the context of an institution like a school or a university, around half the survivors also report the harm of institutional betrayal. Institutional betrayal is the ‘harm that an institution does to those who depend upon it’ [17]. It is evident ‘in overt policies or behaviours, such as discriminatory rules, or an institution’s failure to do what is reasonably expected, including a failure to respond effectively to cases of sexual violence’ [17]. This form of betrayal is very harmful and may exacerbate the physical and emotional symptoms associated with sexual trauma [17].

There are approximately 76 higher education institutions in South Africa, with around two million students and staff across 420 campuses [18]. While there is no data to confirm the true extent of sexual violence in these institutions, a retrospective study on rape justice conducted by the South African Medical Research Council (SAMRC) provides some important insights [19]. The sample for the SAMRC study consisted of 3952 cases of rape reported at 170 police stations across the country in 2012. In these cases, 94.1% of complainants were female, 46% were children (under 18), 4.9% were disabled, and 10.3% of the adult victims were studying at the time [19]. The report does not detail where these reported student rapes occurred or who the perpetrators were. Yet, if one considers the overall sample, one finds that in 69% of the cases, the perpetrator was known to the victim either as an acquaintance (30.4%), a current or ex-partner (13.9%), a relative (10.8%), or in another capacity, such as a teacher, a fellow employee, or a prospective employer (9%) [19].

Some higher education institutions in South Africa have been developing policies to combat sexual violence since the late 1980s [20]. Yet, many still lack a relevant policy framework, and others have failed to implement their policies consistently [19,21]. Consequently, sexual violence remains ‘a key challenge to the post-schooling system’ and female students continue to be plagued by patriarchal practices and sexual violence [22]. Since October 2016, the South African Department of Higher Education and Training (DHET) have been developing a Policy and Strategy Framework to address gender-based violence (GBV). This draft framework could help to address sexual violence on campus [22]. For it to succeed, however, the underlying patriarchal rape culture needs to be challenged since it often undermines the implementation of policies designed to protect students from sexual violence. For example, in 2016, a student was sexually assaulted by a police officer during a protest at a large South African university. However, rather than taking decisive action against the perpetrator and responding to the victim’s trauma, university spokespeople became preoccupied with the wording of their response in the media: ‘Was it a case of rape, or [merely] a case of sexual assault? Did it occur on campus or off campus?’ [23]. Three academics from this university argued that the university’s failure to take decisive action effectively ‘entrenches *rape culture* and dismisses the gravity of the incident and the lasting impact of sexual violence on women’ [23]. Similarly, in 2017, a sexual violence awareness group from another university campus released a statement about the way that their university had responded to two recent incidents of sexual violence. They argued that the university’s official email focused more on ‘condemning the release of the perpetrators’ names’ than providing practical information on how to respond to sexual violence on campus [24]. These responses illustrate how rape culture can undermine justice. 

## 3. Theoretical Framework

### 3.1. Participatory Visual Approach

In this paper, we present results from our ongoing research with students at two South African universities, one in KwaZulu-Natal (KZN) and the other in the Eastern Cape (EC). In our collaboration with these students, we have been using participatory visual methodologies (PVM) to understand and address sexual violence on campus [25]. PVM include a variety of arts-based visual methods, such as collage, photovoice, digital storytelling and participatory video. It is a popular approach when working with youth since it can help to address some of the power imbalances that often emerge between adult researchers and younger participants [26]. PVM can help to articulate information that may otherwise be difficult to share due to illiteracy, language obstacles, or topic sensitivity, such as sexual violence [26,27]. Participatory approaches are aligned with the work of Paulo Freire, and have been credited for bringing marginal or previously-silenced voices to the fore, enabling participants to share their stories and helping to design and deliver interventions informed by their lived experiences [26]. In this study, we combined storytelling with collage making, by asking the students to create a collage and then write a short narrative about it.

### 3.2. Using Transformative Learning Theory

We located the project within Jack Mezirow’s Transformative Learning Theory, which explains learning as a process of development [28]. In line with this framework, participants are viewed as active critical thinkers who are capable of interpreting and reinterpreting their situations and imagining new possibilities [29,30]. By enabling the students to imagine different ways of responding to sexual violence on campus [31], our hope was that the emerging understandings and experiences might contribute to not only breaking the silence that often surrounds such incidents, but also initiating and sustaining critical dialogue aimed at addressing it [32,33]. Through engagement in collage-making and storytelling, we also aimed to examine how the students might imagine alternative organisational structures, interpersonal relationships, cultural milieu, and norms and values in and around the campus [33,34]. Indeed, it is important that students are involved as partners and/or leaders in research so that programs can be more equitable, meaningful, and sustainable [35]. In this study, we engaged students in participatory research in order to explore: (1) What does sexual violence look like on the university campus? And (2) What does it mean to be a woman on campus in the context of sexual violence? 

## 4. Materials and Methods

### 4.1. Our Sites and Participants

In this project, we ran two workshops at two South African universities, which are located in the provinces of the Eastern Cape and KZN, respectively. These two institutions were selected by convenience because this was where two of the leading researchers on the project team were located. Fifteen university students (one male and 14 female), attended our participatory workshops. The participants were all university students in education and shared a common interest in addressing GBV on campus. Yet the two cohorts were contrasted by their degree of familiarity with PVM and their experiences of living in these two provinces. These differences allowed the participants to help co-create knowledge of the multiple realities of GBV and has highlighted the complexities of this phenomenon in our results.

#### 4.1.1. Eastern Cape Workshop

Nine undergraduate student teachers (all female, and all black African) who were registered for, or had just completed, their Bachelor of Education degrees, attended our workshop in the Eastern Cape. All participants belonged to a group known as the Girls Leading Change (GLC), which was formed in 2013. We purposively recruited the GLC members to this project in their first year of study in 2013. They all went to school in rural villages in the Eastern Cape. Over the past five years, the GLC have been using PVM to investigate sexual violence on campus. They have also been using their visual productions to engage university management and community structures in dialogue about sexual violence on campus and on strategies to address it. Since the GLC have been involved in using creative methods to investigate and address sexual violence for some time, we encouraged them to focus on meaning and activism by using the following prompt in our collage and storytelling workshop in 2017: ‘What does it mean to be a young African woman in times of sexual violence?’

#### 4.1.2. KZN Workshop

Six undergraduate students (one male and five females, and all black African) attended the workshop in KZN. These students belong to a self-formed group known as the Gender Activists (GAs) which has been meeting since 2017. GA has twelve members and continues to grow as other interested students join. Our project team meets with the GA once a month to engage in PVM activities with the aim of identifying and addressing sexual violence on campus. In March 2018, we ran a one-day collage and storytelling workshop with six of the GA who were available to attend. Given the mixed-gender make-up of GAs, we decided to use a gender-neutral prompt: ‘What does sexual violence look like on campus? (The face, the act, the space, the emotion)?’ Since the GAs are still quite new to using PVM, the focus of the workshop was more on mapping out the nature of sexual violence on this campus, than on activism.

### 4.2. Using Collage and Storytelling

In both workshops, we employed collage as a method for students to reflect on their experiences of sexual violence on campus, to collaboratively interpret the images, and to devise relevant action plans. Collage creation is known to be a time-consuming process, and yet it is this aspect of the methodology, the fact that it is lengthy, that makes it so suitable for addressing sensitive topics. Indeed, it is this process of cutting, tearing, sticking, drawing, and writing, which allows participants to process their thoughts and feelings and to meditate on the meaning of the issue at hand [36]. We also used storytelling to gain insight into the nature and meaning of sexual violence. Storytelling is a useful tool to apply in the South African context since it has played a central role in our cultural and political history and continues to help marginalized people to share experiences, to listen to each other, and to make sense of their worlds [37,38]. For us in the project, storytelling and the dialogues around the meaning of the collages is a part of breaking the silence and creating critical dialogue about being a female student in South Africa in the time of sexual violence. Indeed, through the collage and storytelling, we were able to engage the students in linguistic and non-linguistic dialogue about what they saw and experienced on campus and how they interpreted it [39,40].

In both workshops, we provided the participants with a prompt, and supplied them with poster materials, various magazines, a pair of scissors, glue and markers, and gave them 120–180 min to respond to the prompt in any way they wanted to. The individuals then wrote down their stories, talked about their individual collages and then we discussed emerging issues across the various collages. With the permission of the participants, the discussions were recorded and later transcribed verbatim.

### 4.3. Data Analysis

The themes that we discuss below emerged inductively through our analysis of the collages, the narratives and our discussions with participants at the workshops. This multi-layered approach to data analysis allowed for the development of a co-constructed understanding of the data.

### 4.4. Ethical Considerations

Ethics approval for this project was obtained from the Research Ethics Committees of the two universities concerned and written informed consent was secured from all participants. In view of the sensitivity of the topic at hand and the prevalence of sexual violence in this population group, we drew on our experience of using these methods to address sensitive topics and took steps to mitigate the risks of re-traumatization and re-victimization [41,42,43]. For example, we created safe spaces by limiting participation to a small group of students who knew each other well. We continuously reminded the participants to refrain from judging or criticizing the content in the collages and what each creator says about their visual creation. As the group’s intention was to potentially use the visual media for advocacy, we specifically chose to use collage as a PVM tool, since it does not reveal personal identities and kept the stories anonymous in our analysis. Lastly, since the process of creating and sharing the stories and collages led to some emotional experiences for some of the participants, we referred a number of them to post-workshop counselling on their respective campuses.

## 5. Findings

Our inductive thematic analysis of these visual texts, stories and discussions resulted in a total number 12 non-exclusive and overlapping themes.

### 5.1. Perspectives from the Gender Activists (GA)

In the KZN workshop, six undergraduates used collage and narratives/stories to explore *what sexual violence looks like on campus*. In Table 1 below, we provide a thumbnail image and brief content description of each of the **six** collages (collages #1 to #6). Our analysis of these artworks, stories, and discussions brought about **seven** nonexclusive and overlapping themes. These were: (1) The Wish List; (2) The Hunt; (3) Rape Gadgets; (4) Standing by my Man; (5) Sex for Favours; (6) My Secret Life; and (7) Agency. We discuss each of these recurrent themes below, drawing on examples from the collages and participant discussions for explanations.

#### 5.1.1. The Wish List

The GAs argued that many young girls come to campus with a ‘Wish List’, expecting to find the ideal person to give them love, material possessions or to fulfil a particular role. As the creator of collage #2 explained, many women come to campus ‘with that sense of freedom’ and the need to be loved by the ‘perfect guy.’ Yet, these expectations can make them more vulnerable to sexual violence since they are fooled by a man’s material possessions and outward charm and fail to recognize when they are in danger. Indeed, one participant explained that behind closed doors a ‘perfect guy’ can transform into a monster: ‘He will tear you up. He will flip in a minute and you will find ‘Mufasa’ [of *the Lion King*] there and you will be behind closed doors’ (creator of collage #3, group discussion).

#### 5.1.2. The Hunt

One participant created a collage (collage #5) with the theme, ‘The Hunt’, in which women are the ‘food on which men prey’. The participant explained that senior male students prey on first year women students and either lure, coerce or force them to have sex (creator of collage #5, group discussion). Picking up on ‘the hunt’ theme, collage #4 refers to ‘rape gadgets’ (discussed further in the section below), which include alcohol, smart cars, cellphones, athletic physiques, and fancy clothes. Men use these ‘gadgets’ to hunt girls and lure and/or force them into sex. This tends to happen at parties, especially during orientation. According to this participant, men watch ‘innocent-looking’ girls arrive at the event and use alcohol to ‘marinate their meat before going in for the kill’ (creator of collage #4, group discussion). For example, one participant explained:

Most of the individuals that come here on campus are from remote [rural] areas that have not really been exposed to this kind of life. So when they see these materialistic things, they automatically just fall for it and then they get taken advantage of. We always want to have fun. We get exposed to life, we get exposed to alcohol and substances and that makes us have poor judgements and that ends up us being getting taken advantage of and getting sexually violated.

(Creator of collage #6, group discussion)

Although alcohol is *officially* prohibited on this campus, the participants explained that the campus security guards generally fail to stop students from drinking on campus and are quite likely ‘to come and join you.’ Yet, at the end of the day, when alcohol ‘turns that guy into a monster’ and he attacks girls in their residence bedrooms or at parties, the campus security guards do not want to deal with the consequences, often blaming the women for drinking and partying and, therefore, for ‘asking for it’(creator of collage #3, group discussion).

#### 5.1.3. Rape Gadgets

As discussed above, some of the collages suggested that men used ‘rape gadgets’ to lure or coerce women to have sex with them. In particular, a few of the collages and the narratives suggest that dress and materialism are closely linked to both ‘the hunt’ and ‘the wish list’ and serve to drive sexual violence on campus. For example, a participant noted, ‘you find that the person comes from a poor family and goes to tertiary education, where they want to fit in in terms of what they should wear and they get this person who will be able to provide for them in terms of the finances, and as to how that person treats them, now that no longer matters.’ According to the participants, social media plays a significant role in policing women’s dress, for example, by showing students ‘what we are supposed to wear, and what we are not supposed to wear, and [influencing] how we get violated as people.’ In collages #2, #4 and #6, and in subsequent discussions, it was clear that female students experienced high rates of cyber violence on campus, where perpetrators plague them in their personal spaces with offensive words and images, such as ‘dick pics.’ Cellphone data is expensive in South Africa and this often limits internet access amongst resource-poor youth. However, on campus, free internet is provided by the university and so student use of social media is high.

According to the participants, men often use their athletic physiques to lure women into having sexual relations with them and then use their physical strength as a weapon to overcome them. collage #5, for example, shows a ‘strong man’—the hunter and the body builder, the perfect guy, who goes to gym. Yet, as the participant argued, the story changes when these guys turn to abuse: ‘I honestly feel they go there to that gym to gym up for us. They are just practicing until they come to this boxing match and they box you because they’ve already practiced how to box a woman’ (creator of collage #3, group discussion). Moreover, when the ‘rape gadgets’ fail to lure women, men often use abuse and violence instead. For example, collage #3 identifies name-calling as another form of gender-based violence that women experience on campus, As the creator of the collage states, the word ‘bitch’ is used pervasively throughout campus to refer to women, ‘you are called the ‘B’ instead of your name.’ Replacing the women’s names with this derogatory word is, according to the participants, a form of abuse, robbing female students of their identities and humanity.

#### 5.1.4. Standing by My Man

Our discussion about dress extended to the link between sexual violence and the kinds of clothes that women wear. For example, one student explained that if a man assaulted her when she ‘was drunk, or wearing revealing clothes, this is seen as a good enough reason for him to go unpunished for his wrong doings’ (creator of collage #1, group discussion). Another student claimed that when other women, friends, police or security personnel witness or receive reports of sexual violence, rather than attempting to help the victim or punish the perpetrator, they choose to ‘stand by their man’ (creator of collage #6, group discussion). They choose to ask: ‘what did you do to get violated? What were you wearing? Why was he in your room? What did you say? Is he your boyfriend? Did you text him first…?’ (creator of collage #3, group discussion). Creator of collage #6 further argued that others try to normalise violence, encouraging the victim to let go of the event and ‘stand by your man.’ Consequently, as creator collage #4 wrote on her artwork, ‘rape is the only crime where the victim becomes the accused.’ Such victim blaming is a serious concern since it leads to apathy and a lack of action. Consequently, leadership and managerial structures on campus often fail to protect students from sexual violence.

#### 5.1.5. Sex for Favours

The collages and discussions also suggest that some of the academic and administrative staff are either complicit in the violence or are perpetrators themselves. Indeed, ‘it seems that if you are given the power to lead, it gives you the right to do as you please to those who do not have power.’ Abuse of power has a major impact on the lives of female students. It influences where they live, since those in charge of housing are known to exchange a room in residence for sexual favours from students. Similarly, lecturers are known to exchange marks/grades for sex. This abuse is illustrated in the image of the two people at the desk in collage #4 and the accompanying text, ‘agree to it and I’ll give you what you need.’ The abuse of power extends to the student leaders from the Students’ Representative Council (SRC), who are said to have:

Taken control of every single aspect of life on this campus [and to] use their leadership skills, alcohol and the privileges that they have to manipulate you as a woman. I feel if you go into their office in order to ask for help, they will sit there, and they will look at you and laugh at you unless you are willing to give them something in order for you to repay them.

(creator of collage #4, group discussion)

This has led our participants to question the leadership on their campus. ‘Not just the students, I question the lecturers, I question the Executive Management for not taking a stand on the situation that we are facing’ (creator of collage #6, group discussion). Yet, as the creator of collage #3 claimed; ‘it seems that people [in power] don’t get punished because they are in the high ranks of the university.’ Moreover, it would seem that justice can be bought, since our participants who have gone to the security structures to report abuse have found that the perpetrators sometimes pay security guards to ensure the sexual violence report disappears. This also happens with the police. ‘The police will come and take statements and when you are supposed to go and face trial, the docket goes missing’ (creator of collage #3, group discussion).

#### 5.1.6. My Secret Life

Fear of abuse and a culture of victim blaming has made it difficult for women to speak about experiences of violence. It has created a culture of silence and secrecy, which results in survivors of sexual violence on campus being isolated. This challenges their dreams of a bright future. As the creator of collage #6 explained:

You spend thousands on your ‘new mattress’ [university and housing fees] only to be violated in this space. You find yourself carrying an unwanted pregnancy and/or HIV. You end up floating on the sea, all alone. The people at home who sent you to study, your families, teachers, and friends, will never find out about your secret.

(creator of collage #6, group discussion)

Creator of collage #6 further notes that ‘we end up with a ‘do not disturb sign’ on the door of our relationships because, even though we are going through problems, we ‘are afraid of what you might find behind the closed door and yet … maybe I can help you to get out of that room’ (group discussion). Thus, the collages also referred to some possible solutions and strategies for addressing sexual violence on campus.

#### 5.1.7. Agency

The collages, in identifying solutions, referred to women’s agency and strength. In some instances, it seems as if there is no hope: ‘we have hindrances as women. Even though we want to help one another, we have things that are hurting us. Things that we cannot do anything about because we are shattered … we don’t have anything else that we can use to empower ourselves’ (creator of collage #3, group discussion). This lack of agency is also apparent in the reports of women forgiving perpetrators without seeking justice. As one participant, who disclosed that she was a survivor of physical violence explained, ‘I don’t hold grudges. I am very forgiving … I wish there could be something I could do, but for me, what has helped is just talking about it’ (unidentified participant, group discussion). 

Yet despite the complications surrounding agency and strength, it is clear that the students believe in themselves and intend to take matters into their own hands. Firstly, one cannot underestimate the importance of agency demonstrated by these students in volunteering to join this group and in their willingness to talk about this problem (with university leadership) and to take joint action. Secondly, the words: ‘the change makers’ in collage #4, confirm this sense of agency since they indicate that ‘it is us who can change the system in order to work for us.’ Moreover, in the group discussion, the creator of collage #6 further explained that the words: ‘question of leadership,’ on her collage are meant to encourage girls to ‘take leadership and question what can we do. What solutions can we take? Where can we seek justice, and find strength.’ Agency is also evident in excerpts from the discussion, such as ‘you go through so much that you can either forget about yourself or it helps you to find the hidden you.’ Or, ‘I wonder what I can do to ensure that cases of sexual violence are dealt with accordingly.’ Lastly, a sense of inner strength is conveyed in the following statement by creator of collage #3: ‘as a South African nation, we fail to understand that even though men are more powerful than us … we also have the power to stand up and say, ‘you know what, I can do better without you.’

### 5.2. Perspectives from the Girls Leading Change (GLC)

Here, we present results from the workshop we held with nine of the 14 GLC members, who created collages and narratives to explore what it means to be a young African woman in times of sexual violence. In Table 2 below, we provide a thumbnail image and brief content description of each of the **nine** collages (collages #7 to #15). Our analysis of the collages, stories and discussions brought about **five** nonexclusive and overlapping themes, including: (1) The Female Body as a Crime Scene; (2) Changing a Man’s World; (3) African Women, Patriarchy, and Change; (4) How to Flourish in Times of Violence; and (5) Social Conscience to Extend the Reach. We discuss each of these recurrent themes below, drawing on examples from the collages, stories, and discussions for illustration.

#### 5.2.1. The Female Body as Crime Scene

This collection of collages offer insight into how young African women feel in times of sexual violence and reveal that although they are ‘made’ in South Africa they do not necessarily feel a sense of belonging or safety in this space. For example, collage #9 features the image of an indigenous African woman dressed in traditional clothes with the caption, ‘Made from Africa.’ Yet, the creator of collage #9 explained, ‘When the world is violent you feel like there is no place to hide, there’s no sense of belonging. … The place where you are supposed to be safe, feels unsafe.’ (Creator of collage #9, group discussion). She continued and pointed out, ‘There is a sense of darkness’ and ‘it is very scary’ (creator of collage #9, group discussion). Feeling scared is linked to bodily harm in the family home, public spaces, cultural practices and coercion. For example, one participant said that young African women do not feel safe when they go out or when they stay home, because their families may sell them off into a forced marriage or they may be raped by their stepfather. Another collage creator explained, women are ‘thrust into an abusive, unjust, uncomfortable, oppressive and dehumanizing situation by persons or circumstances beyond their control,’ which they endure ‘beneath the mask of a smile’ (creator of collage #9, group discussion). Collages #9 and #10 showed the stress, anxiety, and pain beneath the mask. These all limit the potential of women who come to university ‘to start a new life’ (creator of collage #10, group discussion) and to make something of themselves.

Collage #8 depicts a silhouette of the body of an African woman carrying a basket on her head. The collage creator explained that this body is symbolic of the body of African women as ‘a crime scene.’ As the participant explained, ‘I see a woman’s body, from the breast down, from the chest down, as a crime scene… This part of my body is not mine as a woman. As a young African woman, I do not own this part of my body and I am always in a battle for the control and ownership of my body’ (creator of collage #8, group discussion). The participant continued, ‘society lays claim in many ways to the body of women through media, policy, politics, reproductive health choices, domestic control, body shaming… As African women, it is time that we closed that crime scene and said, ‘This is my body. You need… to step out of it and you are not going to tell me what I am.’ (Creator of collage #8, group discussion). This statement illustrates how these young women are taking back ownership of their bodies and are resisting the demands placed on them.

#### 5.2.2. Changing a Man’s World

The impetus to challenge the status quo—a man’s world—is a significant theme in this collection of collages. For example, collage #8 depicts an African woman carrying a bowl on her head, which represents a woman fulfilling her traditional role in society. In the words of the creator of collage #8, it shows ‘what society expects of an African woman’ and that ‘as an African woman, patriarchy has always tried to make sure that [we] conform to the standards and gender roles that our mothers and their mothers have conformed to’ (creator of collage #8, group discussion). Yet collage #9 shows women and girls in a room with a door behind them to depict how some women just ‘kept on knocking until they found the right door’ (creator of collage #9, group discussion). In other words, they persevered until they found a way to participate in the community discussions usually reserved for men. These women saw themselves as able to take charge and ‘speak about things that we are generally not allowed to speak about in African culture’ (creator of collage #8, group discussion). Nevertheless, another participant acknowledged that there were certain constraints within this freedom, ‘you have to follow certain protocols. You have to do certain things. You have to respect their way’ (creator of collage #10, group discussion). Moreover, she continued that they ‘have to grow with so many secrets in [them] because there isn’t much of a space where [they] can just talk about things’ (creator of collage #10, group discussion). 

In the workshop discussions, it was clear that these student teachers saw their role as future teachers as intrinsically important to this vision of transformation, this impetus to change a man’s world. For example, one participant stated that ‘the most important part of being an indigenous African woman in this time is to be a teacher’ (creator of collage #7, group discussion). She further explained that it was important to be ‘a teacher in all aspects, not just a teacher in the classroom.’ She had ‘chosen to teach young men because ‘I feel that when we groom young men, we create a new generation of men who will look at women differently… who will know that it is important to respect a woman…. who will know that you don’t take women for granted.’ Furthermore, this will produce a new generation of young women who ‘know that it is important to value yourself so that other people can value you as well. All this we will do using love’ (creator of collage #7, group discussion).

#### 5.2.3. African Women, Patriarchy and Change

The creator of collage #14 explained that the woman seen gazing into the mirror in her collage is ‘looking at herself. Trying to know who she is, what she is capable of and what her purpose is in life’ (creator of collage #14, group discussion). Living in a man’s world with strict gender prescripts is difficult for indigenous African women. In the words of one participant, ‘Being black. Being a woman. There’s double trouble’ (creator of collage #9, group discussion). Interestingly, some participants stressed that men do not maintain the unequal patriarchal system alone. Women help to preserve it, too, since men are ‘helped by our mothers. They are helped by our sisters. They are helped by other women who continue to perpetuate these standards for women’ (creator of collage #8, group discussion).

Yet one participant emphasized that coming to a university for a higher education qualification gave her ‘a chance to discover that I am much more than I thought I was’ (creator of collage #7, group discussion). For her, coming to university was ‘to start a new life … to make something of myself’ (creator of collage #7). Another participant went on to say that even though indigenous African women are often caught in a net of societal expectations, with courage they can succeed ‘in this time of violence’ (creator of collage #10, group discussion). This, another participant linked to self-belief and agency, as key to this transformation. For example, she stated that ‘part of that change is proactivity or agency on my part as a woman … I need to do something for myself’ (creator of collage #8, group discussion).

A sense of agency and resilience is echoed in collage #13 in the image of a Band-Aid plaster, which, the collage creator explained, shows that one must not ‘let your scars define you’ (creator of collage #13, group discussion). Another participant went on to say that ‘as a young African woman, I feel that part of the change involves defying or redefining gender roles’ (creator of collage #8, group discussion). Moreover, another young woman shared that she is now starting to know who she is. That she has a right to say ‘no and to not do the things that she does not want to do. She also has ‘the right to speak [her] mind, make [her] own decisions’ (creator of collage #13, group discussion). This sense of emancipation and hope is further reflected in the words of another participant, in her ability to ‘depart from all the situations that make us feel like we are trapped in a net. [To] have creative meetings and spaces to help ideas run free. [To] have love and take care of people and … [to] say ‘Now I have my voice. I realize who I am. I realize I have meaning to life’ (creator of collage #10, group discussion).

#### 5.2.4. How to Flourish in Times of Violence

Collage #14 depicts two puzzle pieces with a gap in between them. For this collage creator, the image represents, ‘a person striving to transform into a new person. It is someone trying to connect with this new puzzle piece and trying to … flourish into someone new all over’ (creator of collage #14, group discussion). Yet, this ‘flourishing’ is not always easy, since a person may experience ‘obstacles or challenges when trying to transform’ (creator of collage #14, group discussion). These words are an acknowledgment of the tensions that can arise when one attempts to disrupt societal expectations or when one attempts to challenge traditional gender roles. Another participant posited that to enable the transformation ‘I need to move from all the negatives and expectations that society has of me and my body. I myself need to bring about the change that I want for myself as an African woman’ (creator of collage #8, group discussion). Yet, this cannot be achieved if the ‘lady with a finger on her lips’ depicted in collage #14, ‘is afraid to speak and is afraid of coming out of her comfort zone’ (creator of collage #14, group discussion).

Another participant added that African women need social support and a sense of belonging in order to flourish in these times of violence. ‘As … indigenous African [women] we know how important it is to have a family, to have a sense of belonging, to be who you are and to feel safe where you belong’ (creator of collage #9, group discussion). This idea links to the power of the collective which is reiterated in the following quote from the discussion, ‘[as] women we must have a spirit of unity as I cannot do it alone. We can do it, we can all do it’ (creator of collage #11, group discussion). In addition to social support, one participant also noted that women need knowledge, direction and personal goals in times of violence to ‘know where to go and where you are going, [to have] right choices and goals [and to] be rooted in information as young women’ (creator of collage #10, group discussion). Another participant noted that as an educated woman and as a woman with knowledge about gender inequality and rights ‘I feel like now I have power to control my own life and no one else can control my life. I’ve got the power. Now I have what I call hope. Now I have hope for the future. I have dreams. Now I can make my dreams come true. I have hope that I will become what I want to become’ (creator of collage #13, group discussion).

#### 5.2.5. Social Conscience to Extend the Reach

In the discussion which followed the collage creation, the participants emphasized the importance of sharing what they had learnt with others. For example, drawing on the notion of a woman’s body as crime scene, creator of collage #8 asserted:

I need to clean up my crime scene [and] then I can come up and nurture everybody with all the resources I have in the bowl [reference to image in collage #8 of a basket on the woman’s head] … I have a richness of resources. I can use my independence to protect my world. I can be self-assured and be confident. I can make choices and I can plough that into my community to nurture my community.

(creator of collage #8, group discussion)

This motivation to share knowledge is driven by the need to change the next generation. For example, as one participant stated, ‘the collage represents me passing down all of this to other people, to the children’ (creator of collage #13, group discussion). The power of the collective is further evoked in the following statement, ‘we, as women should stand together. Unite and work together. Rise and not be oppressed by men’ (creator of collage #15, group discussion). This unification and social support is depicted in collage #14, which shows ‘four women holding each other [meaning] that we as women must support each other and we should try to reach out to each other’ (creator of collage #14, group discussion). This choice to offer support and to change society is clear in the following words by one of the participants, ‘I chose to be a lifeline. I chose to be selfless. I chose to help others with what I have learnt so that they can be able to change their lives as well. By choosing to be a lifeline, I chose love as my method’ (creator of collage #7, group discussion).

## 6. Discussion

Our aim in this study was to explore female students’ experiences of sexual violence on campus and to gain insight into the meanings they assign to them. We located the work within transformative learning theory, and used collage and storytelling to help the women students to break the silence around sexual violence and stimulate critical dialogue to address it. Overall, we found that the two participatory workshops allowed the 15 participants a safe space to explore the nature of sexual violence and its meanings to them, and to openly discuss how the problem of rape culture on university campuses might be addressed. The students’ perspectives at both sites point to the fact that sexual violence is entrenched in gender inequality and discrimination, which is deepened by the intersectionality of sex, race, and class [44]. Yet, despite the deep-seated roots of this challenge, the data suggests that, as participatory and transformative approaches, collage-making and storytelling enabled the participants to identify and discuss their agency as women and students and to engage university leadership and their peers in addressing it.

Our use of different prompts at the two workshops and the different backgrounds of the two respective participant groups resulted in there being some striking differences between the data produced in the two settings. For example, while the data from the KZN workshop with the ‘newer’ group of students served mostly to map out ‘what sexual violence looks like’ in this setting, the Eastern Cape workshop with the more experienced group, delved much deeper into the ‘meaning’ of sexual violence against women students and focused on their power and agency as students and future educators in addressing it. Yet despite the differences, in many ways the themes fed into each other, with some notable correlations between the data produced in the two settings. In the next paragraph, we briefly outline the significance of some of the key themes with reference to our aims and relevant literature about this topic.

The data from the KZN workshop with the GAs provides key insight into how rape culture functions in this setting. In many cases, we found clear connections between these findings and research data about rape culture on campuses in other national and international spaces. For example, ‘The Hunt’ theme provided important insight into how and where men perpetrate sexual violence. The link between alcohol and sexual violence is well established in local and international literature [45]. Moreover, it is well known that substance abuse is used to ‘excuse’ the perpetration of sexual violence [46]. Yet, despite this knowledge, it appears that universities do not have strict enough practices to eliminate substance abuse on campus and its use to excuse acts of violence against women. The metaphors of ‘meat’ and ‘the hunt’ and their links to sexual violence shed light on the gender norms that produce violence against women. Meat plays a central role in indigenous cultures of South Africa and is closely linked to wealth, ancestors and power [47]. For example, at most traditional cultural ceremonies, a beast (usually a bull) is slaughtered as a way for the family to connect with, and appease, their ancestors [48,49]. This meat is then cooked and feasted on at the occasion. Traditionally, however, men get the first pickings, followed by the women, and then the children. When viewed metaphorically, as a man’s need for meat is insatiable and comes before the needs of women and children, so too is his need for sex which comes before the wellbeing of others. The data from the Eastern Cape workshop placed greater emphasis on the link between violence and gender norms. This is a key point, since research has shown that there is a ‘strong association between men’s use of violence against women and their beliefs and expectations about men and women’s roles and rights in society’ [50]. Thus, it is time for universities to mobilize their resources and play an active and decisive role in addressing sexual violence by tackling harmful views around gender norms and their links to sexual violence.

The Sex for Favours theme in our data speaks to different forms of transactional sex, which have been identified in recent literature. For example, in a 2016 review of 300 international studies on transactional sex, three basic paradigms were identified, including ‘sex for basic needs,’ which positions women as victims in transactional sexual relationships; ‘sex for improved social status’, which positions women as sexual agents who engage in transactional sex in order to attain middle-class status; and ‘sex and material expressions of love, which draws attention to the connections between love and money, and the central role of men as providers in relationships’ [51]. Echoing available literature, our findings suggest that many women students find themselves abused not only by their student boyfriends, but also by men in power who force them to exchange sex for a particular service, including a place in residence or for marks/grades. This kind of transactional sex can be aligned with the ‘sex for basic needs’ paradigm. The university can address this form of abuse by warning students and staff, equipping them with the skills and knowledge needed to respond effectively to these situations and punishing perpetrators who demand sex for what women students are entitled to, such as a place in residence [36,52]. Our findings also suggest that when women have the courage to report experiences of sexual abuse on campus, the security service often fails to take relevant action against the perpetrator [53]. This is a serious failure in the university’s security system that clearly needs to change. Furthermore, according to our participants, onlookers often choose to ‘stand by the man’ and either blame the women for the abuse or encourage them to forgive the perpetrator and move on [53]. These responses breed a toxic culture of victim blaming, hopelessness, and silence and impunity on the part of the perpetrators. The participants suggest that it is now time for university staff and students to draw on their sense of agency and accountability by adopting a more active stance against sexual violence on campus.

Our findings also speak to the second paradigm, ‘sex for social status’, since, according to our participants, first year students in particular, tend to be easily lured or forced into sex in exchange for material possessions, such as clothes to help them fit in. Women in this category may appear to have more power than in the first paradigm since they have the freedom to choose a partner, but ‘once the choice [is] made, their power [is] greatly circumscribed…’ [54]. This is particularly true with regards to when sex takes place, the nature of the sex and whether condoms are used. These factors have great implications for women’s vulnerability to sexual violence and to sexually transmitted infections, including HIV—with the man changing into a ‘Mufasa’ in the bedroom! The last paradigm also bears connection to our findings, in particular to the ‘wish list’ and rape gadgets themes, which are shown to make women more vulnerable to violence on campus. There is a need to challenge these connections between love and money and the gender normative assumptions of men as providers in relationships.

The data produced in the KZN workshop is also important and can be used by the participants and university leadership to address sexual violence in this setting. The GA plan is to share this data with the university leadership at strategic monthly meetings, and members of the GA are currently using these themes to create improvised dramas about sexual violence on campus. These dramas are followed by student discussions as a way of breaking the silence and finding solutions. Their engagement in this activism links to the final theme of ‘agency’, which links the data from the two settings, with a notable emphasis in the visual and narrative data on the students’ ability to challenge harmful gender norms and change ‘a man’s world.’ For example, the theme, ‘the female body as a crime scene,’ shows a sense of agency through the participants’ determination to regain control of their bodies. So, too, does their use of the word ‘crime,’ which makes it clear that the students are aware that sexual violence is illegal and unacceptable. Moreover, although these students recognize that patriarchal norms remain a challenge, they are clearly determined to address sexual violence in their communities as students and as future educators.

We believe that the GLC’s engagement in this project for the past five years has boosted their sense of individual and collective agency and has primed them to rethink how gender-based violence and sexual violence could be addressed. Indeed, the GLC is well accustomed to using visual methods to explore and address gender-based violence at university [46] and have had several opportunities to engage in dialogue with researchers and university policy-makers [47,48]. The collage and storytelling revealed their deep awareness as university students of the contextual drivers of sexual violence in the broader community, in their communities and on the university campus. The use of PVM, underpinned by feminist assumptions of doing research with marginalized young women, was critical in enabling them to move from normalized lived experience of sexual violence, to awareness of the injustice against them as women and to a conceptual understanding of the structural violence against women. Their engagement in the research over several years, allowed transformative learning to take place, which, as Mezirow (1996) explains, enabled these women to look back, to look inwards, and to look forward to how to address sexual violence in their communities and on campus [28]. It is such transformative learning, we argue, leading to a redefining of the self as women, which can facilitate ‘clos[ing] the crime scene’ and enable women to belong and feel safe in their communities and on campus.

## 7. Conclusions

This paper has illustrated how PVM can be employed to engage with women students and to gain direct insight into what drives sexual violence on university campuses across national and, possibly, international contexts. The students’ perspectives on the nature of sexual violence and possible strategies for addressing it can be harnessed by the leadership of institutions to ground the development and implementation of sexual violence policies on campus. The collages and stories discussed in this paper are reminders that the roots of sexual violence lie in inequality and discrimination and that violent masculinities and acquiescent femininities are often used to normalize sexual violence. Therefore, if sexual violence is to be addressed, we need to challenge gender norms, to stop trying to control and police the behaviour of girls and women, and start interrogating the sexually violent behaviour of boys and men. Our analysis also suggests that PVM can play a key role in this cultural change since the research process can enable deep reflections on the meaning and impact of sexual violence, particularly for women. More importantly, PVM can help young women to break the silence and sustain new conversations about gender and gender equality in institutions and beyond. In 2018, we commemorate 100 years of women’s rights internationally and 24 years of democracy in South Africa and yet the human rights of equality, health, and freedom from violence remain a myth for many women on college and university campuses in South Africa and beyond. As our participants contend, it is time to break the silence about this key issue and to take urgent action to address it.

## Figures and Tables

**Table 1:** Collage descriptions from the KwaZulu-Natal workshop.

No.	Thumbnail	Content
#1	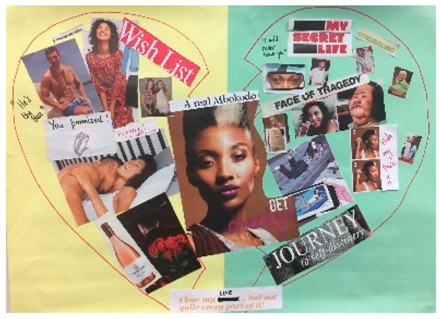	A broken heart frames this artwork. On the left, the words ‘wish list, he’s the best and promise’ are juxtaposed with images of romantic love, such as kissing, flowers, a wedding ring, and a muscular guy. Across the top of the page is written ‘face of tragedy’ and below is a large image of a woman whose face and hair are divided by a birthmark. On the right-hand side, there are images of the same person applying makeup to hide her birthmark and the text reads ‘hide my face, my secret life and journey to self-discovery.’
#2	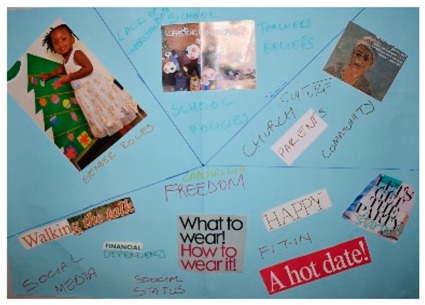	On the top left of this artwork, there is a young girl child wearing a pretty dress and decorating a Christmas tree with text reading, ‘gender roles.’ In the middle, there are school children and the words, ‘school policy, teacher beliefs and lack of understanding.’ On the right, there is a young woman with a headscarf and text, ‘culture church, community and parents.’ At the bottom, there is a lot of text, such as ‘campus life, sense of freedom, social status, social media, need to fit in, a hot date, financial dependency and what to wear.’
#3	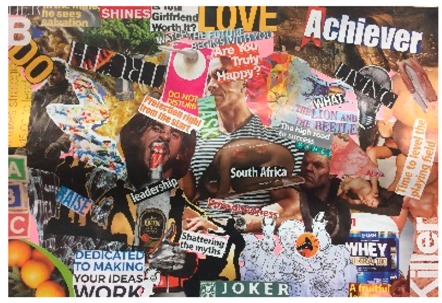	This busy collage features an image of a strong man in the centre with the text ‘are you truly happy?’ Pasted below is an image of the folded body of a naked woman with the names of illnesses pasted on different parts of her body. The text, ‘South Africa’ is pasted over this image. In the top left is the letter ‘B’ and there are multiple images of people, pills, and alcohol, and an image of a woman standing on a man’s face with text, ‘time to level the playing field.’
#4	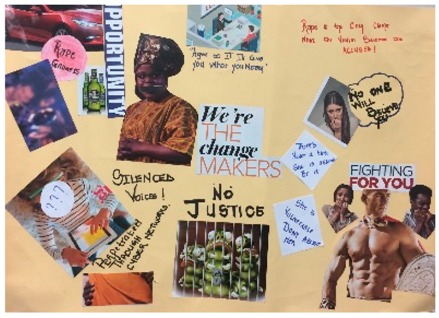	In the centre is ‘We’re the change makers,’ and above are two people at a desk with text, ‘agree to it and I’ll give you what you need.’ Below are some insects in jail. Centre left is an African woman with a taped mouth and ‘silenced voices.’ Above reads, ‘rape gadgets’ with a blurred party image, smart car and alcohol. Below is someone on her tablet, with question marks and ‘perpetuated through cyber violence’. On top right is ‘Rape is the only crime where the victim becomes the accused.’ Below stands a strong man in front of frightened onlookers with words, ‘Fighting for you.’
#5	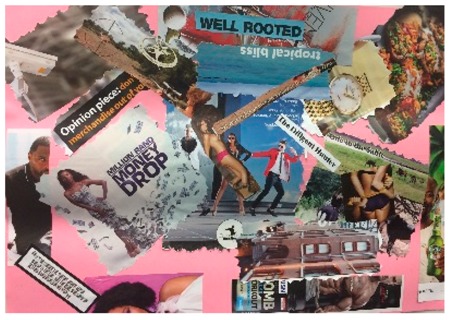	The centre of the collage reads ‘the diligent hunter’ and ‘set your sites on owning a share in this hunting farm.’ Below, two men grab a woman from each side with a naked woman pasted on top of her. Top left is an antelope being hunted and an image of a security camera. Below is woman dressed in money and around the edges of the page are sliced images of people (some with alcohol). At the top are the inverted words, ‘tropical bliss.’ On the right is grilled food and below is an antelope juxtaposed with an image of a woman in underwear whose hands are being tied by a man.
#6	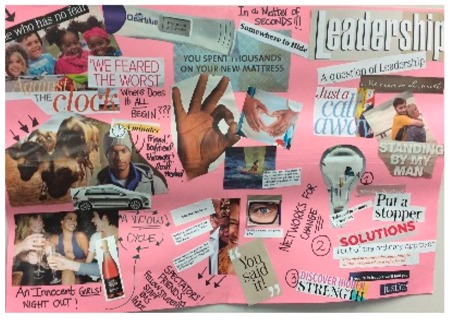	In the centre, is a lonely figure on a canoe with text, ‘I never sent him pictures of my private parts. I might have been drunk and got tired of him asking for my pictures so I just sent them.’ A stained mattress is above with ‘you spent thousands on your new mattress.’ Below is a pregnancy test and ‘somewhere to hide.’ Text bubbles next to three faces say, ‘it happened to us, I am so sorry and I hope that you can see your way to letting this go.’ Arrows point to ‘friends, staff, RMS, police.’ Top left are happy girls and ‘she who has no fear.’ Beneath are men and hunting hyenas and below are young women at a party. Top right reads ‘a question of leadership.’ Below a girl carries a man with text reading ‘standing by my man.’

**Table 2:** Collage descriptions from Eastern Cape workshop.

No.	Collage	Title and Description
#7	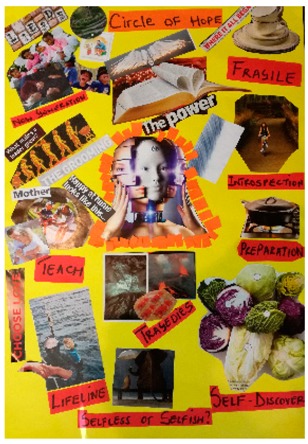	In *Circle of hope*, the text and images tell a story in a circular process. Starting on the right, there is an image of a porcelain cup and saucer symbolizing a ‘fragile’ woman. There is a collection of pictures and text, including ‘introspection’ with a woman alone on her bicycle and ‘preparation’ with a pot on a fire. Text, including ‘lifeline’, ‘teach’, ‘love’, ‘mother’, and ‘new generation’ are juxtaposed with images of families and children. In the centre, there is an image of a woman and a volcano next to the word ‘tragedies’ and another image of a Bible, together with the word ‘power.’
#8	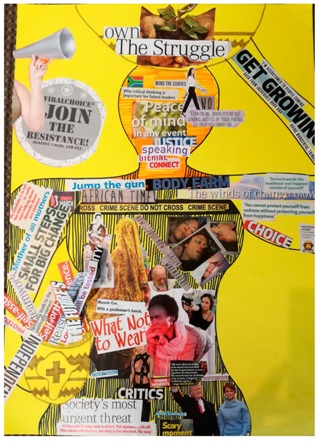	*Transcendence* features the shape of a woman’s body filled with images and text about resistance and sexual violence. On the left, there is a loudhailer and text which reads, ‘join the resistance’, and on the right, there is text which reads ‘get growing’. The woman carries a traditional basket on her head with text reading, ‘own the struggle.’ On her face, there is an image of a woman walking tall and the words ‘peace of mind’, ‘justice’, and ‘why critical thinking is important for future leaders.’ Lines of text, such as, ‘crime scene do not cross’, divide the woman’s head from her body.
#9	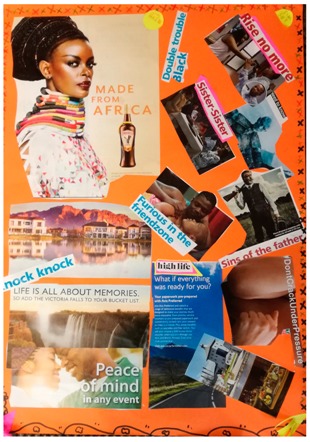	*My smile is my story* has an African woman on the right, with the words ‘made from Africa.’ On the left, there are images of violence, such as a woman pointing a gun and a man with a gun over his shoulder and text ‘the sins of the father.’ Between these images are the words, ‘double trouble Black’, and below is a group of women sitting around a table, accompanied by the text ‘sister sister.’ Beneath that, words read, ‘don’t crack under pressure’, and there is a picture of a road and the words, ‘high life.’ On the bottom left, a couple embrace with the text ‘peace of mind’.
#10	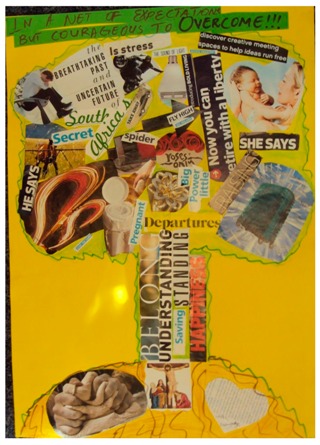	*In a net of expectations, but courageous to overcome* is in the form of a tree. The roots section has images of a cross, bread dough and a heart. The trunk reads, ‘belonging,’ ‘happiness’ and ‘understanding.’ On the left of the canopy, text reads, ‘he says’, and a man is sitting with his head in his hands, juxtaposed with the text, ‘breathtaking past and uncertain future.’ In the middle is a rider falling off his motorbike and the words, ‘stress’, ‘spider and others.’ On the right of the canopy are the words, ‘she says’ and there is picture of a mother and baby with, ‘now you can live with a liberty’ and ‘discover creative meeting spaces to help ideas run free.’
#11	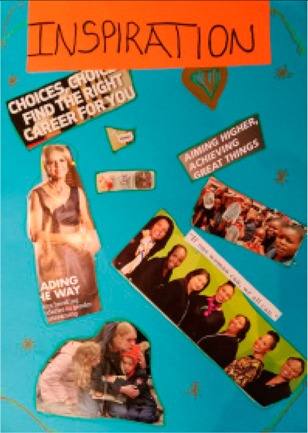	*Inspiration* showcases many images of women, including a single woman, a woman with her two children, a group of women and a group of children. There is also text reading, ‘leading the way,’ ‘choices choices, find the right career for you’ and ‘aiming higher, achieving great things’.
#12	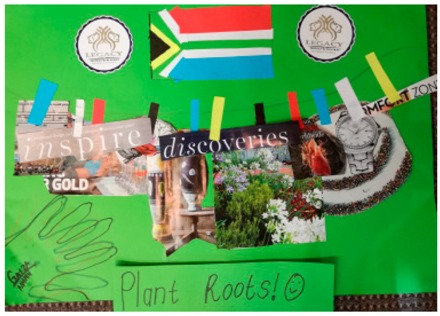	*Plant Roots* is presented in the form of a clothes line. A row of colourful pegs secures a variety of images to the line, including a watch, flowers and vases. There is also text hanging on the line, such as ‘discoveries’, ‘inspire’ and ‘comfort zone.’ A South African flag and two circles with the text ‘legacy’ on them are at the top of the collage. At the bottom left hand corner, the artist has traced her hand and signed her first name.
#13	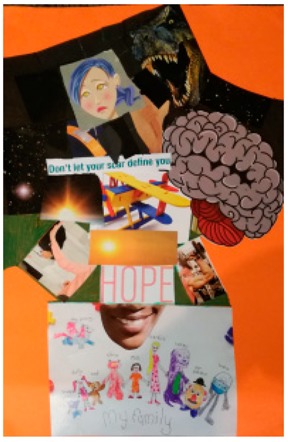	*There’s still hope,* is divided into a black section in the upper half, and a white section below. These two sections are filled with various images and text about sexual violence. For example, in the dark part, there is a face of a fearful woman surrounded by a dinosaur with an open mouth displaying sharp teeth. There is also the muscular arm of a man and a brain, with text saying, ‘don’t let your scars define you.’ On the white section, below, there is a woman’s smiling mouth above a drawing of a big family, with the word, ‘HOPE’ written in a large font across the centre.
#14	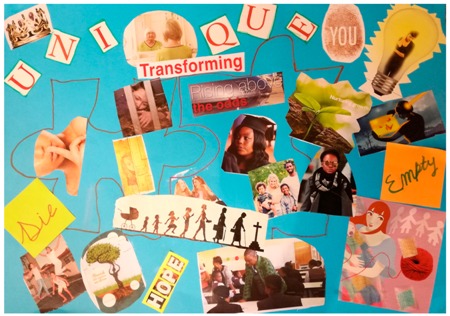	*Transforming* displays two puzzle pieces with a gap in between them. In the centre is the text, ‘transforming and an image depicting the development of woman from cradle to grave. Other images include a woman looking into a mirror, a thumb print, a woman in a lightbulb, a teacher, an older woman, a woman graduate and a woman behind bars, and one with her finger on her lips. Prominent text includes the words, ‘hope’, ‘unique’, and ‘rising above the odds.’ There are two images of nature, with the accompanying text, ‘nurturing growth’ and ‘flourish into someone new all over.’
#15	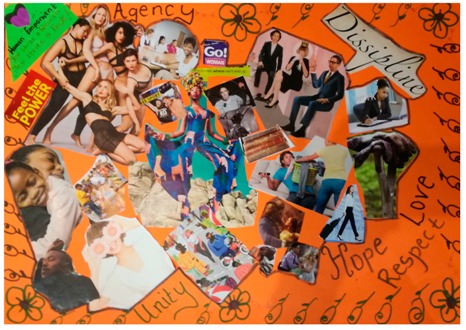	*Women Empowerment* is a busy collage comprising a collection of images and text, including images of women: single women; women in groups; women and men and a woman with a child. One image shows a man with a basket of laundry and another is of a pile of books. The text which surrounds these images are words such as ‘agency,’ ‘love,’ ‘respect,’ ‘hope,’ ‘unity,’ ‘discipline,’ ‘feel the power’, and ‘go woman’.

## References

[1] Krug E.G., Dahlberg L.L., Mercy J.A., Zwi A.B., Lozano R. (2002). World Report on Violence and Health.

[2] Krebs C., Lindquist C., Berzofsky M., Shook-Sa B., Peterson K., Planty M., Langton L., Stroop J. (2016). Campus Climate Survey Validation Study Final Technical Report.

[3] Du Toit L., Gouws A. (2005). A phenomenology of rape: Forging a new vocabulary for action. (Un)thinking Citizenship.

[4] Africa Check: Sorting Fact from Fiction. https://africacheck.org/factsheets/factsheet-south-africas-201516-crime-statistics/.

[5] Kapps C. (2006). Rape on trial in South Africa. Lancet.

[6] Naidoo K. (2013). Rape in South Africa—A call to action. S. Afr. Med. J..

[7] Knox C., Monaghan R., Stanko E. (2003). Violence in a changing political context: Northern Ireland and South Africa. The Meanings of Violence.

[8] Jewkes R., Abrahams N. (2002). The epidemiology of rape and sexual coercion in South Africa: An overview. Soc. Sci. Med..

[9] Moffat H. (2006). These women, they force us to rape them: Rape as a narrative of social control in post-apartheid South Africa. J. S. Afr. Stud..

[10] Moynihan M.M., Banyard V.L., Cares A.C., Potter S.J., Williams L.M., Stapleton J.G. (2015). Encouraging responses in sexual and relationship violence prevention: What program effects remain 1 year later?. J. Int. Violence.

[11] Phipps A., Smith G. (2012). Violence against women students in the UK: Time to take action. Gend. Educ..

[12] Jewkes R. Gender-based violence and HIV: What don’t we know? In Proceedings of the Higher Education and Training HIV/AIDS Programme; Gender & Health Research Unit, Medical Research Council: Pretoria, South Africa, 2017.

[13] Bennett D., Elyse B.A., Guran L., Michelle B.A., Ramos C., Margolin G. (2011). College students’ electronic victimization in friendships and dating relationships: Anticipated distress and associations with risky behaviors. Violence Vict..

[14] UNICEF—The United Nations Entity for Gender Equality and the Empowerment of Women (UN Women), The United Nations Population Fund (UNFPA), The International Labour Organisation (ILO), The Office of the Special Representative of the Secretary-General on Violence against Children (OSRSG/VAC) (2013). Breaking the Silence on Violence against Indigenous Girls, Adolescents and Young Women: A Call to Action Based on an Overview of Existing Evidence from Africa, Asia Pacific and Latin America.

[15] World Health Organization/London School of Hygiene and Tropical Medicine (2010). Preventing Intimate Partner and Sexual Violence against Women: Taking Action and Generating Evidence.

[16] Kaminer D., Grimsrud A., Myer L., Stein D.J., Williams D.R. (2008). Risk for post-traumatic stress disorder associated with different forms of interpersonal violence in South Africa. Soc. Sci. Med..

[17] The Conversation: Academic Rigour, Journalistic Flair. http://theconversation.com/when-sexual-assault-victims-speak-out-their-institutions-often-betray-them-87050.

[18] Department of Higher Education and Training (DHET) (2016). DHET Appoints Team to Address SGBV in HE Institution.

[19] Mercilene M., Jina R., Labuschagne G., Vetten L., Loots L., Swemmer S., Meyersfeld B., Jewkes R. (2017). Rape Justice in South Africa: A Retrospective Study of the Investigation, Prosecution and Adjudication of Reported Rape Cases from 2012.

[20] Hames M., Beja N., Kgosimmele T., Bennett J. (2005). The impact of sexual harassment policies in South African universities: The University of the Western Cape. Killing a Virus with Stones? Research on the Implementation of Policies against Sexual Harassment in Southern African Higher Education.

[21] Joubert P., Rothmann S., van Wyk C. (2011). The effectiveness of sexual harassment policies and procedures at higher education institutions in South Africa. SA J. Hum. Resour. Manag..

[22] South African Department of Higher Education and Training DHET (2017). Addressing Gender-Based Violence in the Post-School Education and Training Sector, Draft Policy and Strategy Framework.

[23] The Daily Vox Team UKZN Fails in Its Concern for Survivors of Rape and Sexual Assault. 8 September 2016. https://www.thedailyvox.co.za/ukzn-response-rape-survivor/].

[24] UCT Survivors (2017). Statement RE: SRC Suspensions. https://uctsurvivors.wordpress.com/2017/07/12/statement-re-src-suspensions/.

[25] Mitchell C., De Lange N., Moletsane R. (2017). Participatory Visual Methodologies Social Change, Community and Policy.

[26] Mitchell C., Sommer M. (2016). Participatory Visual Methodologies in Global Public Health. Glob. Public Health.

[27] Gubrium A., Fiddian-Green A., Lowe S., DiFulvio G., Del Toro-Mejías L. (2016). Measuring down: Evaluating digital storytelling as a process for narrative health promotion. Qual. Health Res..

[28] Mezirow J. (1996). Contemporary paradigms of learning. Adult Educ. Q..

[29] Lynn M. (2016). Backpacking Brothers: An Experiential, Adventure Education Program to Transform Rape Culture and Prevent Sexual Violence. Master’s Thesis.

[30] Lawrence C., Mhlaba T., Stewart K.A., Moletsane R., Gaede B., Moshabela M. (2017). The Hidden Curricula of Medical Education: A Scoping Review. Acad. Med..

[31] Gibbs A., Jewkes R., Mbatha N., Washington L., Willan S. (2014). Jobs, food, taxis and journals: Complexities of implementing Stepping Stones and Creating Futures in urban informal settlements in South Africa. Afr. J. AIDS Res..

[32] Ngidi N.D., Moletsane R. (2015). Using transformative pedagogies for the prevention of gender-based violence: Reflections from a secondary school-based intervention. Agenda.

[33] Meyers S.A. (2008). Using transformative pedagogy when teaching online. Coll. Teach..

[34] Harrell-Levy M.K., Kerpelman J.L. (2010). Identity process and transformative pedagogy: Teachers as agents of identity formation. Identity Int. J. Theory Res..

[35] Dills J., Fowler D., Payne G. (2016). Sexual Violence on Campus: Strategies for Prevention.

[36] Butler-Kisber L. (2010). The power of visual approaches in qualitative inquiry: The use of collage making and concept mapping in experiential research. J. Res. Pract..

[37] Scheub H. (2010). The Uncoiling Python: South African Storytellers and Resistance.

[38] Greene E., Del Negro J. (2010). Storytelling Art and Technique.

[39] Mayaba N., Wood L. (2015). Using drawings and collages as data generation methods with children: Definitely Not. Child’s Play. Int. J. Qual. Res..

[40] Van Schalkwyk G.J. (2010). Collage life story elicitation technique: A representational technique for scaffolding autobiographical memories. Qual. Rep..

[41] Treffry-Goatley A., Wiebesiek L., de Lange N., Moletsane R. (2017). Technologies of Non-violence: Ethical Participatory Visual Research with Girls. Girlhood Stud..

[42] Treffry-Goatley A., Wiebesiek L., Larkin R., Ngcobo N., Moletsane R. (2016). Ethics of Community Based Participatory Research in Rural South Africa: Gender Violence through the Eyes of Girls. Learn. Landsc..

[43] Treffry-Goatley A., Moletsane R., Wiebesiek L. (2018). “You are Welcome, Just Don’t Change Anything”: Challenges and Opportunities in Engaging Communities in Participatory Visual Research to Address Violence against women and girls in rural South Africa. Youth Engagement through the Arts and Visual Practices to Address Sexual Violence.

[44] Jordan-Zachery J.S. (2007). Am I a Black Woman or a Woman Who Is Black? A Few Thoughts on the Meaning of Intersectionality. Polit. Gend..

[45] Sisonke Gender Justice Wits-Sonke Study Reveals Alarming Levels of Men’s Violence against Women in Diepsloot. http://genderjustice.org.za/news-item/wits-sonke-study-reveals-alarming-levels-mens-violence-women-diepsloot/.

[46] Reid A., Dunded L. (2017). Bystander Programs: Accommodating or Derailing Sexism?. Behav. Sci..

[47] Edwards S., Makunga N., Thwala J., Mbele B. (2009). The role of the ancestors in healing: Indigenous African healing practices. Indilinga Afr. J. Indig. Knowl. Syst..

[48] Bogopa D. (2010). Health and ancestors: The case of South Africa and beyond. Indo Pac. J. Phenomenol..

[49] Ainslie A. (2014). Harnessing the ancestors: Mutuality, uncertainty and ritual practice in the eastern cape province, South Africa. Int. Afr. Inst..

[50] De Lange N., Mitchell C., Moletsane R. (2015). Critical Perspectives on digital spaces in educational research. Perspect. Educ..

[51] Stoebenau K., Heise L., Wamoyi J., Bobrova N. (2016). Revisiting the understanding of transactional sex in sub-Saharan Africa: A review and synthesis of the literature. Soc. Sci. Med..

[52] Jones K. (2009). Sexual Assault on Campus: Barriers Curb Reporting on Campus Sexual Assault.

[53] Dastile N.P. (2008). Sexual victimization of female students at a South African tertiary institution: The victim’s perception of the perpetrator. Acta Criminol. S. Afr. J. Criminol..

[54] Jewke R., Morrell R. (2012). Sexuality and the limits of agency among South African teenage women: Theorising femininities and their connections to HIV risk practices. Soc. Sci. Med..

